# A multi-element psychosocial intervention for early psychosis (GET UP PIANO TRIAL) conducted in a catchment area of 10 million inhabitants: study protocol for a pragmatic cluster randomized controlled trial

**DOI:** 10.1186/1745-6215-13-73

**Published:** 2012-05-30

**Authors:** Mirella Ruggeri, Chiara Bonetto, Antonio Lasalvia, Giovanni De Girolamo, Angelo Fioritti, Paola Rucci, Paolo Santonastaso, Giovanni Neri, Francesca Pileggi, Daniela Ghigi, Maurizio Miceli, Silvio Scarone, Angelo Cocchi, Stefano Torresani, Carlo Faravelli, Christa Zimmermann, Anna Meneghelli, Carla Cremonese, Paolo Scocco, Emanuela Leuci, Fausto Mazzi, Massimo Gennarelli, Paolo Brambilla, Sarah Bissoli, Maria Elena Bertani, Sarah Tosato, Katia De Santi, Sara Poli, Doriana Cristofalo, Michele Tansella

**Affiliations:** 1 Section of Psychiatry, Department of Public Health and Community Medicine, University of Verona and Azienda Ospedaliera Universitaria Integrata Verona, Verona, Italy; 2 Agenzia Sanitaria e Sociale Regionale, Regione Emilia Romagna, Verona, Italy; 3 IRCCS Centro S.Giovanni di Dio Fatebenefratelli, Brescia, Italy; 4 Department of Mental Health, Azienda ULSS, Bologna, Italy; 5 Department of Psychiatry, University of Padova, Padova, Italy; 6 Department of Mental Health, Azienda ULSS, Rimini, Italy; 7 Department of Mental Health, Azienda ULSS, Florence, Italy; 8 Department of Mental Health, Azienda ULSS S. Paolo, Milan, Italy; 9 Department of Mental Health, Azienda Ospedaliera Ospedale Niguarda Ca’ Granda, Milan Programma 2000, Italy; 10 Department of Mental Health, Azienda ULSS, Bolzano, Italy; 11 Department of Psychology, University of Firenze, Florence, Italy; 12 Department of Mental Health, Azienda ULSS, Parma, Italy; 13 Department of Mental Health, Azienda ULSS, Modena, Italy; 14 DISM, Inter-University Center for Behavioural Neurosciences, University of Udine, Udine, Italy; 15 Section of Psychiatry, Department of Public Health and Community Medicine, University of Verona and Azienda Ospedaliera Universitaria Integrata Verona, Piazzale L.A. Scuro 10, 37134, Verona, Italy

**Keywords:** First-episode psychosis, Early psychosis, Cognitive behavioral therapy, Psychosocial intervention, Assertive community treatment, Family intervention

## Abstract

****Background**:**

Multi-element interventions for first-episode psychosis (FEP) are promising, but have mostly been conducted in non-epidemiologically representative samples, thereby raising the risk of underestimating the complexities involved in treating FEP in ‘real-world’ services.

****Methods/Design**:**

The Psychosis early Intervention and Assessment of Needs and Outcome (PIANO) trial is part of a larger research program (Genetics, Endophenotypes and Treatment: Understanding early Psychosis - GET UP) which aims to compare, at 9 months, the effectiveness of a multi-component psychosocial intervention versus treatment as usual (TAU) in a large epidemiologically based cohort of patients with FEP and their family members recruited from all public community mental health centers (CMHCs) located in two entire regions of Italy (Veneto and Emilia Romagna), and in the cities of Florence, Milan and Bolzano. The GET UP PIANO trial has a pragmatic cluster randomized controlled design. The randomized units (clusters) are the CMHCs, and the units of observation are the centers’ patients and their family members. Patients in the experimental group will receive TAU plus: 1) cognitive behavioral therapy sessions, 2) psycho-educational sessions for family members, and 3) case management. Patient enrolment will take place over a 1-year period. Several psychopathological, psychological, functioning, and service use variables will be assessed at baseline and follow-up. The primary outcomes are: 1) change from baseline to follow-up in positive and negative symptoms’ severity and subjective appraisal; 2) relapse occurrences between baseline and follow-up, that is, episodes resulting in admission and/or any case-note records of re-emergence of positive psychotic symptoms. The expected number of recruited patients is about 400, and that of relatives about 300. Owing to the implementation of the intervention at the CMHC level, the blinding of patients, clinicians, and raters is not possible, but every effort will be made to preserve the independency of the raters. We expect that this study will generate evidence on the best treatments for FEP, and will identify barriers that may hinder its feasibility in ‘real-world’ clinical settings, patient/family conditions that may render this intervention ineffective or inappropriate, and clinical, psychological, environmental, and service organization predictors of treatment effectiveness, compliance, and service satisfaction.

****Trial registration**:**

ClinicalTrials.gov Identifier NCT01436331

## **Background**

It has been suggested that most clinical and psychosocial deterioration in psychosis occurs within the first 5 years of illness onset, and that this timeframe is a crucial period for initiating treatment [1]. Recent research efforts have therefore focused on early detection and intervention for psychosis, showing that the beneficial effects of antipsychotic medication on first-episode psychosis (FEP) are tempered by the fact that, despite initial symptom reduction, functional recovery is typically poor even when optimal pharmacological treatment is provided [2]. Family members are also affected by the emotional burden of being caregivers, and often show signs of psychological distress themselves [3]. It is clear from the literature that pharmacotherapy alone is not sufficient to prevent relapses or assure functional recovery from acute psychosis [4].

Over the past few years, there has been a growing interest in psychosocial intervention as a means of facilitating recovery and reducing long-term disability associated with psychosis [5]. Literature on psychosocial interventions in FEP can be viewed in terms of two broad categories [6]: 1) studies evaluating specific (that is, single-element) psychosocial interventions (for example, individual cognitive behavioral therapy), and 2) studies evaluating comprehensive (that is, multi-element) interventions, which may include: early detection strategies; individual, group, and/or family therapy; and case management (in addition to pharmacological treatment). These interventions appear promising [7] and have been found to be associated with symptom reduction/remission, improved quality of life, increased social and cognitive functioning, low inpatient admission rates, improved insight, high degree of satisfaction with treatment, less time spent in hospital, decreased substance abuse, and fewer self-harm episodes.

However, most multi-element research programs have been conducted in non-epidemiologically representative samples in experimental settings, thereby raising the risk of underestimating the complexities involved in treating FEP in ‘real-world’ services [8]. Moreover, these interventions have rarely been tested for their efficacy against a control group, but more typically against historical or prospective comparison groups, and usually only single-group studies have been carried out, which track the progress of a single group over a given period.

With respect to clinical practice, some countries have implemented specific early psychosis interventions over the past 10 years, but even these have not yet become routine [9]. Few studies have identified barriers that may hinder the feasibility of these interventions or the patient or family conditions that may render this type of treatment ineffective or inappropriate. Hence, efforts to implement multi-element interventions targeted to FEP in routine services should be accompanied by rigorous scientific method, with the aim of better understanding the actual effectiveness of this approach [10,11].

## **Methods/Design**

### **Aims**

The Psychosis early Intervention and Assessment of Needs and Outcome (PIANO) trial is part of the larger research program termed Genetics Endophenotypes and Treatment: Understanding early Psychosis (GET UP; national coordinator: Professor Mirella Ruggeri, Verona), funded by the Italian Ministry of Health as part of a National Health Care Research Program (Ricerca Sanitaria Finalizzata) coordinated by the Academic Hospital of Verona (Azienda Ospedaliera Universitaria Integrata Verona).

GET UP consists of four partner projects : PIANO, TRaining and Understanding of service Models for Psychosis Early Treatment (TRUMPET), Genetic data Utilization and Implementation of Targeted drug Administration in the clinical Routine (GUITAR) and COgnitive Neuroendophenotypes for Treatment and RehAbilitation of psychoses: Brain imaging, InflAmmation and StresS (CONTRABASS). Each of these partner projects pertains to different areas of research, but they are linked together.

The GET UP PIANO trial described here is the main data collection axis for the overall GET UP Research Program. The trial has three aims:

1) To compare, at 9 months, the effectiveness of a multi-component psychosocial intervention with that of treatment as usual (TAU) in a large epidemiologically based cohort of patients with FEP and their family members recruited from a 10 million-inhabitant catchment area.

2) To identify the barriers that may hinder its feasibility in real-world routine clinical settings and patient/family conditions that may render this intervention ineffective or inappropriate.

3) To identify clinical, psychological, environmental, and service organization predictors of treatment effectiveness, compliance, and service satisfaction in FEP in the Italian community mental healthcare framework.

Study participants are recruited from community mental health centers (CMHCs) operating for the Italian National Health Service and located in two entire regions of Italy (Veneto and Emilia Romagna), and in the cities of Florence, Milan and Bolzano. For administrative and research purposes, the overall territory covered by the GET UP has been divided into eight macro-areas, named participating units (PUs): Western Veneto, Eastern Veneto, Emilia, Romagna, Florence, Bolzano, Milan Niguarda, and Milan San Paolo.

### **Design**

The PIANO trial has a pragmatic cluster randomized controlled design [12,13], which compares the effectiveness of TAU plus a multi-element psychosocial treatment for patients with FEP and their family members, versus TAU alone, as provided by Italian community mental health services. A cluster design was chosen based on feasibility considerations, supported by the MRC Health Services and Public Health Research Board [14], which indicated that cluster randomization is the gold standard approach for trials evaluating similar complex interventions implemented at the institutional level, with the aim of improving health. The assignment units (clusters) are the CMHCs located in the catchment area, and the units of observation and analysis are the CMHC patients and their family members. Each CMHC belongs to the Department of Mental Health (DMH), which is responsible for all mental health care (including outpatient, inpatient, and long-term residential care) for a specific catchment area. Each DMH can include one or several CMHCs, depending on the size of the DMH catchment area.

### **Inclusion and exclusion criteria**

Inclusion criteria are based on the screening method adopted in the WHO 10-country study [15], and include:

· Age 18–54 years.

· Residence in the catchment area of participating CMHCs.

· Presence of 1) at least one of the following symptoms: hallucinations, delusions, qualitative speech disorder, qualitative psychomotor disorder, bizarre or grossly inappropriate behavior; or 2) at least two of the following symptoms: loss of interest, initiative and drive, social withdrawal, episodic severe excitement, purposeless destructiveness, overwhelming fear, marked self-neglect, as measured by the Screening Schedule for Psychosis [15].

· First lifetime contact with participating CMHCs, prompted by the symptoms enumerated in the point above.

Exclusion criteria are:

· Pre-existing anti-psychotic medication (> 3 months) prescribed by any psychiatric or other medical agencies for a mental disorder identical or similar to the current one.

· Mental disorders due to a general medical condition.

· Moderate to severe mental retardation as determined by clinical functional assessment.

· An ICD-10 diagnosis other than F20-F29, F30.2, F31.2, F31.5, F31.6, F32.3, F33.3, F1x.4; F1x.5, F1x.7, F1x.8, F1x.9, as confirmed after 9 months by using the Schedule for Clinical Assessment in Neuropsychiatry (SCAN) [16].

### **Ethical issues**

This study is being conducted in accordance with globally accepted standards of good clinical practice, in agreement with the Declaration of Helsinki, and in keeping with local regulations.

GET UP PIANO investigators ensure that all professionals involved in the trial are adequately qualified and informed about the protocol, the study interventions, and their trial-related duties and functions. The coordinating center maintains a list of all appropriately qualified persons involved in the study.

#### ***Ethics committee approval***

Formal ethics approval for conducting the trial has been sought and obtained by the Coordinating Center’s Ethics Committee (Comitato Etico per la Sperimentazione, Azienda Ospedaliera di Verona, http://www.ospedaliverona.it/Istituzionale/Comitati-Etici/Sperimentazione), which approved the study protocol, the information sheets (patient and family versions), and the informed consent sheets (patient and familiar versions) on 6 May 2009 (Prot. N. 20406/CE, Date 14/05/2009), and by the ethics committee of each participating unit.

### **Interventions**

#### ***Experimental treatment***

The experimental treatment package is provided by routine public CMHCs, which operate within the Italian National Health Service and consists of TAU (see next paragraph) plus evidence-based additional treatment. Specifically, the additional treatment comprises three main forms of intervention: 1) cognitive behavioral therapy for psychosis (CBTp) for patients; 2) family intervention for psychosis (FIp); and 3) case management (CM).

CBTp is based on the model developed by Kuipers *et al.*[17], Garety *et al.*[18] and Fowler *et al.*[19], and the model has already been evaluated in randomized controlled trials [20]. It is expected that an optimal number of 20–30 CBT sessions per patient will be delivered during a time frame of 9 months, with weekly sessions held during the first 3 months and fortnightly during the following 6 months. Family intervention is based on the model proposed by Leff *et al.*[21] and further developed by Kuipers *et al.*[22]. It consists of an optimal number of 10–15 sessions over 9 months with each individual family: 6 sessions in the first 3 months, and at least 1 session/month during the following 6 months. For case management, every patient/family has a dedicated case manager, who coordinates all planned interventions.

Experimental interventions are expected to begin as soon as the patient is stabilized (clinical stabilization is defined as a condition allowing the patient to collaborate in at least a brief clinical examination) and after he/she has been assessed with the ‘core’ set of baseline measures (see ‘Baseline assessment’ below). Before the start of the trial, professionals using the experimental interventions received specific training programs in CBTp (conducted as part of the scientific aims of the GET UP PIANO Project), FIp, and CM (conducted as part of the scientific aims of the GET UP TRUMPET Project). At the end of the training, an assessment of the competence achieved was performed, and detailed intervention manuals based on international standards were developed and given to the professionals as a standard to be followed for the treatment. Professionals are supported in their clinical work by a team of expert psychotherapists assigned to each CMHC. Moreover, experimental interventions provided to all patients/relatives are supervised by a team of external experts who hold one-day meetings every 2 months and are regularly available for consultation.

Fidelity will be measured at the end of the trial by an independent team by using audiotape recordings of therapy sessions, and therapists ratings of their own session. The Cognitive Therapy Scale-Revised (CTRS) [23] and the Cognitive Therapy for Psychosis Adherence Scale (CTPAS) [24] will be used, together with *ad hoc* checklists based on the specific trial intervention manuals, in accordance with the method described in Mc Hugo *et al.*[25].

#### ***Treatment as usual***

TAU is also provided by routine public CMHCs operating within the Italian National Health Service, as above. Standard care for patients with FEP typically consists of personalized outpatient psychopharmacological treatment, combined with non-specific supportive clinical management at the CMHC level. Family interventions generally consist of non-specific informal support/educational sessions. Specialized individual psychotherapeutic interventions (including CBT) for patients and specialized psychoeducational or cognitive-oriented family interventions are usually not provided because of the lack of trained professionals [26,27].

### **Enrollment procedure for community mental health centers**

The GET UP PIANO trial catchment area covers 126 CMHCs (9,951,306 inhabitants), all of which were officially asked to participate in GET UP; 117 agreed to participate, covering a catchment area of 9,304,093 inhabitants. In an effort to improve the efficiency of the study design, CMHCs were stratified before randomization, based on three variables: affiliation to the same DMH, CMHC catchment area size, and type of area (urban/mixed versus rural). Socioeconomic levels in the trial catchment area were not considered as stratification variables as they were found to be reasonably homogeneous. With the exception of staff members in five of the CMHCs (covering a catchment area of 503,000 inhabitants) the mental health staff of the remaining 112 CMHCs had received no previous training in the intervention. These first 5 centers were therefore ‘forced’ into the intervention arm and used as the expected ‘gold standard’ in the analysis, in order to measure the competence of the remaining professionals. Thus, 112 CMHCs (8,801,093 inhabitants) were available for the randomization procedure. Because of organizational needs, 32 small CMHCs were paired, based on their affiliation to the same community psychiatric service, thus resulting in 16 randomization units. Hence, in total, 96 units entered the randomization procedure.

### **Enrollment procedure for patients and family members**

The CMHCs participating in the study are asked to refer all potential cases of psychosis at their first contact with the service over a period of 1 year. The Screening Schedule for Psychosis [15] is administered to all patients as soon as possible after first service contact, and must be administered within 15 days. Inclusion criteria are based on the screening methodology adopted in the WHO 10-country study [15].

In both experimental and control CMHCs, patients meeting all inclusion criteria are invited to undertake standardized assessments as soon as possible once they achieve clinical stabilization (see ‘Baseline assessment’ paragraph). Eligible patients are also asked for consent to involve a key family member in the assessments. An independent, trained researcher conducts the informed consent interview, as approved by the ethics committee of the Coordinating Center at the Academic Hospital of Verona and the local ethics committees. All participants are informed that it is possible to withdraw consent to assessments at any time. If the patient or the family member does not agree to be assessed, the independent researcher briefly records the reasons for refusal, whenever possible. Once informed consent is obtained, independent researchers complete the baseline quantitative assessments for both the participant and the family member. As a minimum, the ‘core’ set of baseline assessments (see ‘Baseline assessment’) are completed before the beginning of treatment (either TAU plus experimental therapy or TAU alone). If the patient is not accessible for baseline assessment (consent given but appointments missed, lack of time, etc.), the interventions begin if possible with the same time schedule, and the core baseline measures are retrospectively reconstructed by consulting the patient’s case record.

From 1 May 2011, a checking procedure using both screening and baseline assessment phases is being undertaken in the CMHCs participating in the study, in order to guarantee the completeness and the accuracy of the recruitment procedure and to identify any missed cases. Generally, this procedure is called the ‘leakage study’ and is considered a fundamental part of pragmatic epidemiologically based trials. All electronic and paper information systems in the CMHCs are carefully scrutinized for any cases aged 18–54 years, presenting to the services for the first time during the index period, ICD-10 diagnostic codes of psychosis (F20-F29, F30.2, F31.2, F31.5, F31.6, F32.3, F33.3, F1x.4; F1x.5, F1x.7, F1x.8, F1x.9). These data are compared with case records to confirm eligibility. This procedure will be completed on 31 January 2012. All identified patients will therefore be invited to participate in the informed consent interview, and the ‘core’ baseline measures (see ‘Baseline assessment’) will be retrospectively reconstructed by consulting the patient’s case record.

### **Baseline assessment**

After clinical stabilization, patients are assessed by independent evaluators with a set of standardized instruments (Table 1) to measure: premorbid IQ (Italian version of the New Adult Reading Test; Test Intelligenza Breve (TIB)) [28], substance abuse (Clinical Drug Use Scale; CDUS) [29], symptoms (Positive and Negative Syndrome Scale; PANSS) [30], (Hamilton Rating Scale for Depression; HAMD) [31], (Bech-Rafaelsen Mania Rating Scale; BRMRS) [32]], global functioning (Global Assessment of Functioning; GAF) [33], subjective appraisal of positive symptoms (Psychotic Symptom Rating Scale; PSYRATS) [34], social disability (Disability Assessment Scale; DAS) [35], insight (Schedule of Assessment of Insight; SAI-E) [36], needs for care (Camberwell Assessment of Needs; CAN-EU) [37], quality of life (World Health Organization Quality of Life; WHOQOL-Bref) [38], life events (first 14 years of life, 1 year before the onset of psychosis and period after onset; *ad hoc* schedule for life events [39]; Childhood Experience of Care and Abuse Questionnaire (CECA-Q) [40]), parental bonding (Parental Bonding Instrument; PBI) [41], and expressed emotions (Level of Expressed Emotion Scale; LEE) [42]. The ‘core’ set of baseline assessments includes PANSS, HAM-D, BRMRS, GAF, and DAS. Patients are also assessed by a set of tests evaluating neuropsychological performance (such as Wisconsin Card Sorting Test (WCST), AX-Continuous Performance Test (AX-CPT), and Wechsler Adult Intelligence Scale (WAIS)).

**Table 1:** Study schedule: instruments used at baseline and follow-up assessments in the Genetics, Endophenotypes and Treatment: Understanding Early Psychosis (GET UP) Psychosis Early Intervention and Assessment of Needs and Outcome (PIANO) trial

	**Enrolment (12 months)**	**Baseline**	**Follow-up (9 months from baseline)**
Patients			
Review of inclusion criteria	X		
Informed consent signed	X		
Consent for involvement of family members	X		
Sociodemographic and clinical data		X	
Instruments			
Core set: PANSS, HAM-D, BRMRS, GAF, DAS		X	X
TIB, Life events (CECA-Q, PBI), neuropsychological tests (WCST, AX-CPT, WAIS)		X	
CDUS, PSYRATS, SAI-E, CAN-EU, WHOQOL-Bref, LEE		X	X
LCS, VSSS-EU			X
SCAN			X
Psychosocial/pharmacological treatment/admissions schedule			X
VITreT			X
Reporting of pharmacological side-effects and other adverse events		X	X
Family members			
Informed consent signed	X		
IEQ-EU, GHQ		X	X
PSA		X	
VSSS-Relatives			X

AX-CPT, AX-Continuous Performance Test; BRMRS, Bech-Rafaelsen Mania Rating Scale; CAN-EU, Camberwell Assessment of Needs; CDUS, Clinical Drug Use Scale; CECA-Q, Childhood Experience of Care and Abuse Questionnaire; DAS, Disability Assessment Scale; GAF, Global Assessment of Functioning; GHQ, General Health Questionnaire; HAM-D, Hamilton Rating Scale for Depression; IEQ-EU, Involvement Evaluation Questionnaire; LCS, Life Chart Schedule; LEE, Level of Expressed Emotion Scale; PANSS, Positive and Negative Syndrome Scale; PBI, Parental Bonding Instrument; PSA, Premorbid Social Adjustment; PSYRATS, Psychotic Symptom Rating Scale; SAI-E, Schedule of Assessment of Insight; SCAN, Schedule for Clinical Assessment in Neuropsychiatry; TIB, Test Intelligenza Breve (Italian version of the New Adult Reading Test); VITreT, Verona Interview for Treatment Termination; VSSS-EU, Verona Service Satisfaction Scale, patient version; VSSS-Relatives, Verona Service Satisfaction Scale, relatives version; WAIS, Wechsler Adult Intelligence Scale; WCST, Wisconsin Card Sorting Test; WHOQOL-Bref World Health Organization Quality of Life.

Participating patients are asked for consent to involve their family members in the trial. If this is given, family members are also asked to provide written informed consent, and are assessed about their burden of care (Involvement Evaluation Questionnaire; IEQ-EU) [43] and emotional distress (General Health Questionnaire; GHQ) [44]. They are also interviewed about the patient’s premorbid adjustment (Premorbid Social Adjustment scale; PSA) [45].

Before starting the assessments, the independent evaluators receive formal training in the use and administration of the instruments, with measurement of their skills and knowledge, and assessment of inter-rater reliability.

### **Follow-up assessment**

After a 9-month period from baseline assessment, patients are reassessed to measure: substance abuse (CDUS) [29], symptoms (PANSS [30], HAMD [31], BRMRS [32]), global functioning (GAF) [33], subjective appraisal of positive symptoms (PSYRATS) [34], social disability (DAS) [35], insight (SAI-E) [36], need for care (CAN-EU) [37] and quality of life (WHOQOL-Bref) [38] (Table 1). Patients are also evaluated in terms of pharmacological side-effects and other adverse events, pattern of clinical course (Life Chart Schedule; LCS) [46] and service satisfaction (Verona Service Satisfaction Scale, patient version; VSSS-EU) [47].

Family members are reassessed with respect to burden of care (IEQ-EU) [43] and emotional distress (GHQ) [44], and are also assessed for service satisfaction (VSSS, relatives version; VSSS-Relatives) [47].

The formal best-estimate research diagnosis is assessed at the 9-month follow-up using the item group checklist of the SCAN [16]. All relevant baseline and follow-up information is obtained and reviewed by two independent raters to formulate the ICD-10 diagnosis. In cases where a consensus is not reached, the opinion of a third rater is solicited to clarify diagnostic problems.

Psychosocial and pharmacological treatment, together with number and days of admission provided in the 9-month follow-up period, are recorded in a detailed *ad hoc* schedule.

All patients who have terminated contact with the service before the 9-month follow-up are traced and asked to undergo a semi-structured interview on the characteristics of treatment termination (Verona Interview for Treatment Termination, VITreT). This consists of 10 questions assessing: if the decision to interrupt service contacts was shared or not with the key clinician; the reason for interruption; which type of assistance was eventually received thereafter in other settings, including admission to hospital; and satisfaction with the care provided in these settings [48].

### **Randomization procedure**

**Figure 1:**
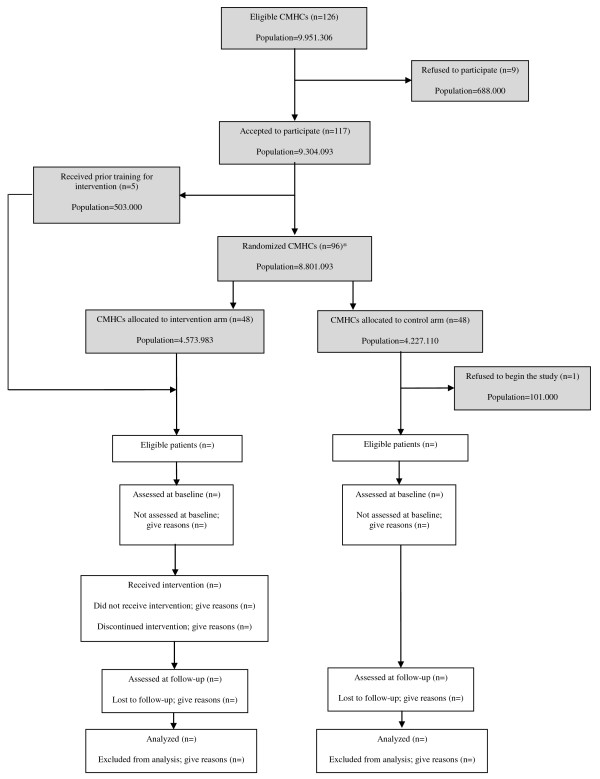
Flow diagram of Community Mental Health Centers (CMHCs) and patients in the Genetics, Endophenotypes and Treatment: Understanding early Psychosis (GET UP) Psychosis early Intervention and Assessment of Needs and Outcome (PIANO) trial.

In total, 96 units entered the randomization procedure, with equal numbers being allocated to each arm. These units were randomly assigned to one of the two trial arms with a 1:1 allocation rate. The trial statistician (blind to CMHC identity) prepared the sequence of treatments (experimental treatment versus TAU) randomly permuted into blocks of two. The randomization schedule was generated using Stata software (version 11.0; Stata Corp, Corp., College Station, TX, USA), using the ‘ralloc’ command for random allocation of treatments balanced in blocks. Subsequently, arm allocation was disclosed to each CMCH, and the allocation sequence was not altered. One CMHC refused to begin the study immediately after the randomization procedure (Figure 1).

Eligible patients were assigned to treatment (experimental intervention versus TAU) in accordance with the allocation of CMHC pertaining to the catchment area of residence.

### **Primary outcomes**

The primary outcomes are:

· Changes from baseline to the 9-month follow-up assessment in positive and negative symptom severity and subjective appraisal, as measured by the positive and negative subscales of the PANSS and by the PSYRATS.

· Relapse occurrences during the period between baseline and the 9-month follow-up assessment, where relapse is defined as an episode that has resulted in an admission to a psychiatric inpatient unit (number and days of hospitalization) and/or any case noted record of re-emergence after a period of full/partial remission of positive psychotic symptoms of at least moderate degree requiring a significant change in the clinical management (for example, increased visiting or medication levels) [49]. Consensus ratings will be made by paired members of the research team (blind to the randomization arm) using *a priori* operational definitions.

In this pragmatic trial, two primary outcome measures were defined in order to detect more finely-tuned clinical changes also in those patients (nearly 60%) [50] who are not expected to relapse over the study period, and in those who have a continuous course of illness.

In addition to relapse occurrences, the number of months in full or partial remission will be calculated in a randomly selected subsample of 30% of the participating subjects. This will be obtained by means of a published method for rating remission in psychosis that has been used in previous randomized controlled trials [18,49,50]. Consensus ratings will be made by paired members of the research team using manualized *a priori* operational definitions, a method with good validity and moderate to good reliability [18,49]. Ratings are based on the level of positive psychotic symptoms, and the technique will be applied to detailed extracts of the clinical case notes. These will consist of monthly reports over 9 months on mental state and service interventions, from which all information identifying the location of the clinical service and the nature of treatment will have been removed. Actual group allocation will remain concealed until all ratings are complete. Data on all hospital admissions will be retrieved from the hospital discharge records administrative database.

### **Secondary outcome measures**

The secondary outcomes will be:

· change from baseline to 9 month follow-up in patient functioning, assessed using the GAF and the WHO-DAS;

· change from baseline to 9 month follow-up patient emotional wellbeing, measured by using the anxiety and depression items of the PANSS and the HAM-D and selected items of the WHOQOL-Bref;

· service disengagement and time to service disengagement, assessed by consulting case records and local databases;

· change from baseline to 9 month follow-up in patient needs for care, assessed using the CAN

· change from baseline to 9 month follow-up in expressed emotions of the key relative, measured using the LEE

· change from baseline to 9 month follow-up in burden of the key relative, measured using the IEQ

· service satisfaction in patients and relatives, measured using the VSSS-EU and VSSS-Relatives scales.

### **Reporting and quantification of side-effects**

No specific side-effects are expected from the interventions being tested. Monitoring and registration of death (from any cause), suicide attempts, serious medication side-effects (neuroleptic malignant syndrome, tardive dyskinesia, akathisia, and tremors) for participants in both treatment arms will be maintained over the study duration. Across both arms, these adverse events will be recorded by the treating psychiatrists as they occur and by the assessors who collect data at 9 months.

### **Sample size and power calculations**

For power calculations, we consider rates of relapses and/or severe psychotic symptoms to be the primary outcome measures. For a conventional trial with randomization of individual patients [51], a total of approximately 250 patients will detect a difference in terms of rates at 9 months from 25% in the TAU arm to 10% in the experimental treatment arm, with a power of 80% (two-sided test at 5%, http://statpages.org/proppowr.html). This difference could represent a plausible and realistic intervention effect [7,52,53]. The cluster randomization used for the GET UP PIANO trial purposes might result in reduced efficiency and loss of power because the within-cluster responses tend to be more similar than those of individuals from different clusters (commonalities in selection, exposure, shared environment, mutual interaction). A larger sample size will therefore be needed to compensate for this clustering effect. The clustering effect is measured as 1 + (*m*−1)*ρ*, where *m* is the number of participants per cluster (assuming equal sizes for clusters) and *ρ* the intra-cluster correlation coefficient (ICC). This clustering effect is used as an inflation factor to increase the sample size calculated, as required by an individual randomization trial [51,54]. Our approach is simplified because it does not take into account variations in the number of participants in each cluster and assumes *m* to be the average number of participants per cluster. Although this type of imbalance in cluster size may reduce the power of the trial, the loss is negligible for studies with more than 100 patients per arm [55]. Based on the additional assumptions of an ICC of 0.05 and an average of four eligible and consenting patients in each cluster, the number of patients required would be approximately 350. With a loss to follow-up of approximately 10%, we expect that a sample size of about 400 patients will yield sufficient power. Assuming an expected incidence rate in Italy for non-affective psychoses of 11 per 100,000 per year [56] and a rate for affective psychoses rate of 6 per 100,000 per year [57], a reasonable estimate of the number of patients expected over 1 year from the 116 participating CMCHs (total population 9,203,093; at-risk population about 50%) is about 800. Assuming an attrition rate of approximately 50%, due to a number of reasons, both at the cluster level (drop-out of CMHCs from the study, lack of cooperation) and at the individual level (participants who do not seek help, do not attend the public services, refuse to be involved in the study), the number of patients available for the trial would be about 400.

With regard to family members, the key-relative burden assessed by using the IEQ was used as the outcome measure for power calculation. From previous studies [58,59], we expect the mean reduction of total IEQ score to be 8.0 in a total score range of 27 to 135 for the family members allocated to TAU, with a standard deviation of 15.0. We want to be able to detect at least a 50% greater reduction in the burden in the experimental intervention group at the 0.05 level of significance and with a power of 80%. For a conventional trial with randomization of individual subjects, a sample size of 280 will allow detection of this difference in key-relative burden. By applying the inflation factor to account for the cluster design, the sample size required would increase up to 320. Based on the expected number of patients (n = 400), it seems reasonable to assume that a 20% of family members will not be traceable, will refuse to participate, or will drop out from the trial. Thus, the expected number of about 300 family members would be sufficient to detect a significant difference in burden.

### **Blinding**

In this pragmatic trial, implemented in the CMHC settings that are the randomization units of the trial, the blinding of patients, clinicians, and raters working on site is not possible. However, every effort will be made to preserve the independence of the raters. They are not involved in the treatment sessions, and any conflict of interest is accurately prevented and monitored. In any case, possible bias associated with lack of blinding is expected to have limited effect on the estimates of intervention effects because the primary outcomes (relapses and/or changes in psychopathology) are objective clinical assessments [60,61]. Moreover, for the assessment of primary outcomes, raw data will be analyzed whenever possible by paired and independent members of the research team who are blinded to the randomization arm.

### **Types of analysis**

All study data are entered into an electronic database and stored at the World Health Organization Collaborative Center for Research and Training in Mental Health and Service Evaluation of the University of Verona. The trial data manager is not involved in determining patient eligibility, administering treatment, or determining outcomes. A set of electronic and manual edit checks ensures data correctness and consistency. In accordance with the Declaration of Helsinki, patient confidentiality is fully preserved during all the study phases via anonymous data recording. Patients are assigned an identification number, both in the baseline and follow-up forms, and in the database. Anonymized data will be transferred to the trial statistician for the analyses.

Data will be analyzed when information is available for all participants. The pattern of missing values will be explored, and interactions between treatment group and completers/non-completers will be examined.

Statistical analysis will be based on an intention-to-treat (ITT) basis, comparing outcomes from all patients within CMHCs allocated to experimental treatment with those allocated to TAU. The emphasis will be on differences between these groups in terms of changes from the pre-intervention to the post-intervention phases, by taking appropriate account of the clustering. The ITT principle will allow for potential biases arising from loss to follow-up, under the assumption that missing outcomes were missing at random (MAR) using the terminology of Little and Rubin [62].

Owing to the characteristics of the cluster randomization study design, the statistical analysis cannot be masked, that is, the trial statistician will not be blinded to the treatment groups, although he/she will not be involved in determining patient eligibility, in administering treatment, in measuring outcomes, or in entering data. All analyses will be performed using Stata software (11.0 for Windows; Stata Corp.).

### **Statistical analysis**

Findings will be reported in accordance with the CONSORT guidelines for cluster randomized trials [63,64]. The nesting of different units of observation (patients) in each unit of assignment (CMHCs) and in different units of experimental condition assignment (experimental treatment versus TAU) will create the hierarchical structure that is characteristic of cluster randomized trials. Patients will be the first level, CMHCs the second level, and treatment assignment the third level of aggregation.

Firstly, the baseline characteristics of patients and clusters will be compared to ensure effective randomization. To compare differences in outcomes between experimental treatment and TAU, we will use an analysis appropriate for cluster randomized trials [65], namely, a *t*-test weighted by an inverse binomial variance weight for binary outcomes, and a *t*-test weighted by an inverse variance for continuous outcomes [66]. The key is to assess the variation of the chosen condition-level summary statistic (for example, mean or proportion) against the variation of the corresponding group-level statistic through the use of weights proportional to the inverse of the variances of the cluster means or proportions. Regarding multilevel analyses, Murray *et al.*[13] reviewed model-based methods appropriate for the cluster randomized design. Random-effects regression models [67] will be used to compare treatment outcomes between the study groups because they include all sources of random variation, and reflect regression adjustment for covariates at both individual and cluster level. The ‘gllamm’ command in Stata 11.0 will be used for this purpose. The effects of baseline covariates expected to have an important influence on the primary outcome variables will be controlled for, by comparing covariate-adjusted analyses with unadjusted analyses. Specifically, important covariates for the outcome are gender, duration of untreated psychosis, and age of onset. The presence of multicolinearity, interaction, and higher power terms will be assessed to check final model validity. Mixed models will allow for the inclusion of data from patients with incomplete observations at follow-up. We will allow for the presence of missing outcome data under the assumption that the data are missing completely at random, conditional on the covariates included in the models (that is, MAR, using the terminology of Little and Rubin [62]). Statistical significance will be defined at two-sided *p* < 0.05. All analyses will be performed using Stata software (version 11.0 for Windows; Stata Corp).

### **Planned subgroup analyses**

The primary outcome may vary in subgroups of patients with different baseline characteristics. Consequently, secondary analyses will be carried out to compare the outcome in groups of patients with specific characteristics identified *a priori* (such as gender, age of onset, duration of untreated psychosis), so as not to pose multiplicity concerns. Power for these subgroup analyses has not been specifically allowed for, and so they will be treated as exploratory, and will not affect the trial’s conclusions. Planned subgroup analysis will be performed by using the ITT approach, based on subgroups [68].

### **Informed consent form and information sheet**

Eligible participants are asked to participate only after receiving a detailed explanation of the nature, scope, and possible consequences of the trial. Participants receive an informed consent document including both information about the study and the consent form to sign. This document contains all the elements required by the *Guidelines of Good Clinical Practice* and any additional elements required by local regulations. The document is in a language understandable to the participants and specifies the person (either a psychiatrist or a psychologist) who informs the participant. After reading the informed consent document, the patient or their legal representative gives consent in writing. The patient’s consent is confirmed at the time of the consent by the personally dated signature of the participant and by the personally dated signature of the person conducting the informed consent discussion. In accordance with the *Guideline of Good Clinical Practice*, participants enrolled in the trial with the consent of the participants’ legally acceptable representative are informed about the trial to the extent compatible with the participants’ understanding and, if capable, the participant is asked to sign and personally date the written informed consent.

Participating patients are asked to give consent for the involvement of their family members in the study, and those providing consent receive an informed consent document that includes both information about the study and the consent form, which is given to family members. The staff member or the researcher informing family members is a psychiatrist or a psychologist. After reading the informed consent document, the participant gives consent in writing.

### **Independent data-monitoring committee**

The trial is regulated by an independent trial monitoring committee including experts who hae reviewed and approved the protocol before commencing enrolment. Adverse events will be monitored and discussed with this committee.

### **Trial status**

The trial began on 1 April 2010 and is still ongoing. The patient enrolment will finish on 31 January 2012, and the follow-up assessments are expected to be completed on 31 May 2012.

## **Discussion**

Psychotic disorders are the most severely disabling of all mental illnesses, leading to great personal suffering for patients and their family members, due to still-persisting social stigma and repeated post-relapse hospitalizations. Most clinical and psychosocial deterioration in schizophrenia has been found to occur within the first 5 years of illness onset, suggesting this phase as a “critical period” for initiating treatment. Thus, the most recent research applications in the field have begun to focus on the aspects of early detection and intervention, with findings now revealing a direct relation between quality of clinical/social response and swiftness of treatment after psychosis onset. International treatment guidelines for first episode psychosis now recommend a prompt and integrated pharmacological and psychosocial approach, including cognitive behavioural psychotherapy for patients and psycho-educational intervention for their family members. Hence, policy planning must also be based on a combination of these different components in a multi-element perspective. However, there is little knowledge on how these procedures can be best integrated into current CMHC clinical practices. The challenge is therefore that of learning how to effectively manage many inter-dependent organisational problems and to concurrently develop and implement intervention programmes that are targeted, effective, and tailored to patients and their family members. Moreover, all of this must be achieved in a context of great (patient, family, clinical, and social-relational) variability.

The Research Programme “Genetics Endophenotypes and Treatment: Understanding early Psychosis” (GET-UP) aims to apply innovative and targeted forms of early psychosis onset intervention and to test its effectiveness and feasibility in Italian Community Mental Health Centres. The randomised controlled trial launched in the frame of the GET UP Research Programme, whose protocol is described in this paper, is based on sophisticated epidemiological, clinical, biological, and neurocognitive investigations and involves 117 Mental Health Centres located throughout a 10 million-inhabitant catchment area, including two Regions (Veneto and Emilia Romagna) and the Bolzano, Florence, and Milan provinces. Workers in these catchment areas are trained in the above-mentioned forms of intervention. This initiative is expected to produce scientific knowledge useful to activate a virtuous circle to foster the dissemination of early prevention and intervention practices--not only for psychoses, but also in other mental health spheres.

## **Abbreviations**

AX-CPT, AX-Continuous Performance Test; BRMRS, Bech-Rafaelsen Mania Rating Scale; CAN-EU, Camberwell Assessment of Needs; CBT, Cognitive Behavioral Therapy; CDUS, Clinical Drug Use Scale; CECA-Q, Childhood Experience of Care and Abuse Questionnaire; CM, Case management; CMHC, Community Mental Health Centre; CONSORT, Consolidated Standards Of Reporting Trials; CONTRABASS, Cognitive Neuroendophenotype for Treatment and Rehabilitation of Psychoses, Brain Imaging, Inflammation and Stress; CTPAS, Cognitive Therapy for Psychosis Adherence Scale; CTRS, Cognitive Therapy Scale-Revised; DAS, Disability Assessment Schedule; DMH, Department of Mental Health; FEP, First-episode psychosis; FI, Family Intervention; GAF, Global Assessment of Functioning; GET UP, Genetics Endophenotypes and Treatment Understanding Early Psychosis; GHQ, General Health Questionnaire; GUITAR, Genetic Data Utilization and Implementation of Targeted drug Administration in the Clinical Routine; HAMD, Hamilton Rating Scale for Depression; ICC, Intra-cluster correlation coefficient; ICD, International Classification of Diseases; IEQ-EU, Involvement Evaluation Questionnaire; IQ, Intelligence Quotient; ITT, intention-to-treat; LCS, Life Chart Schedule; LEE, Level of Expressed Emotion; MAR, missing at random; PANSS, Positive and Negative Syndrome Scale; PBI, Parental Bonding Instrument; PIANO, Psychosis early Intervention and Assessment of Needs and Outcome; PSA, Premorbid Social Adjustment; PSYRATS, Psychotic Symptom Rating Scale; PU, Participating unit; SAI-E, Schedule of Assessment of Insight; SCAN, Schedule for Clinical Assessment; TAU, Treatment as usual; TIB, Test Intelligenza Breve; TRUMPET, Training and Understanding of service Models for Psychosis Early Treatment; VITreT, Verona Interview for Treatment Termination; VSSS-EU, Verona Service Satisfaction Scale; WAIS, Wechsler Adult Intelligence Scale; WCST, Wisconsin Card Sorting Test; WHO, World Health Organization; WHOQOL-Bref, World Health Organization Quality of Life.

## **Competing interests**

The authors declare that they have no competing interests.

## **Authors’ contributions**

MR devised the training for the cognitive behavioral intervention prior to the start of the trial; conceived the trial aims and design, the overall epidemiological and training frame, the methodology of study data collection, the monitoring of local sites, and the procedures for data analyses; carried out the literature search, wrote the draft manuscript and finalized the final version. CB, the statistician, contributed to defining the study design, overall methodology, and the procedures for data analyses; planned the statistical procedures; performed the literature search; and contributed to the writing and revision of the manuscript. AL contributed to the concept of trial aims and design, the overall epidemiological frame, the methodology of study data collection, and the procedures for data analyses; contributed to devising the strategies for local data collection in the Veneto Region; performed the literature search; and contributed to the writing and revision of the manuscript. GDG devised the training for the family intervention prior to the start of the trial; contributed to devising the overall epidemiological and training frame, the methodology of study data collection, and the strategies for local data collection in the Emilia Romagna Region; performed the literature search; and contributed to the revision of the manuscript. AF devised the training for the case-management intervention prior to the start of the trial; contributed to devising the overall epidemiological frame, the methodology of study data collection, and the strategies for local data collection in the Emilia Romagna Region; and revised the final version of the manuscript. PR contributed to devising the overall epidemiological frame, the methodology of study data collection, and the strategies for local data collection in the Emilia Romagna Region; and contributed to the revision of the manuscript. GN contributed to devising the overall epidemiological frame, the methodology of study data collection, and the strategies for local data collection in the Emilia Romagna Region; and revised the final version of the manuscript. FP contributed to devising the overall epidemiological frame, the methodology of study data collection, and the strategies for local data collection in the Emilia Romagna Region; and revised the final version of the manuscript. DG contributed to devising the training for the case management intervention prior to the start of the trial; contributed to devising the overall epidemiological frame, the methodology of study data collection, the strategies for local data collection in the Emilia Romagna Region; and revised the final version of the manuscript, P Santonastaso contributed to devising the overall epidemiological frame, the methodology of study data collection, and the strategies for local data collection in the Veneto Region; and contributed to the revision of the manuscript. MM contributed to devising the overall epidemiological frame, the methodology of study data collection, and the strategies for local data collection in Florence; and contributed to the revision of the manuscript. SS contributed to devising the overall epidemiological frame, the methodology of study data collection, and the strategies for local data collection in Milano; and contributed to the revision of the manuscript. AC contributed to devising the training for the experimental interventions prior to the start of the trial, the overall epidemiological frame, the methodology of study data collection, and the strategies for local data collection in Milano; and revised the final version of the manuscript. S Torresani contributed to devising the overall epidemiological frame, the methodology of study data collection, and the strategies for local data collection in Bolzano; and revised the final version of the manuscript. CF devised the methodology for the life events assessments; contributed to devising the overall epidemiological frame and the methodology of study data collection; and revised the final version of the manuscript. CZ contributed to devising the overall epidemiological and training frame, the methodology of study data collection, and the strategies for the data collection in the local sites; and revised the final version of the manuscript. AM devised the training for the cognitive behavioral approach prior to the start of the trial, the supervision of the experimental treatments in the local sites, the overall epidemiological frame, the methodology of study data collection, and the strategies for local data collection in Milano; performed the literature search; and revised the final version of the manuscript. CC contributed to devising the strategies for local data collection in the Veneto Region, and revised the final version of the manuscript. P Scocco contributed to devising the strategies for local data collection in the Veneto Region, and revised the final version of the manuscript. EL contributed to devising the strategies for local data collection in the Emilia Romagna Region, and revised the final version of the manuscript. FM contributed to devising the strategies for local data collection in the Emilia Romagna Region, and revised the final version of the manuscript. MG contributed to devising the overall epidemiological and training frame, the methodology of study data collection, and the strategies for the data collection in the local sites (with specific reference to the neurocognitive and biological assessments); and revised the final version of the manuscript. PB contributed to devising the overall epidemiological and training frame, the methodology of study data collection, and the strategies for the data collection in the local sites (with specific reference to the neurocognitive and biological assessments); and revised the final version of the manuscript. SB, MEB, STosato, KDS, SP contributed to devising the overall epidemiological frame, the methodology of study data collection, and data management, to the training of the team of expert psychotherapists assigned to each CMHC, and to the planning of their work in the local sites; and revised the final version of the manuscript. DC conceived the data-management methodology; contributed to devising the overall epidemiological frame, the methodology of study data collection, and the monitoring in the local sites; and revised the final version of the manuscript. MT contributed to conceiving the trial aims and design, the overall epidemiological and training frame, and the methodology of study data collection; and contributed to the writing and revision of the manuscript. All authors read and approved the final manuscript.

## **Funding**

Italian Ministry of Health, Ricerca Sanitaria Finalizzata *(National Health Care Research Program),* CUP Code 61 J08000200001.
